# Transformer Fault Diagnosis Based on Knowledge Distillation and Residual Convolutional Neural Networks

**DOI:** 10.3390/e27070669

**Published:** 2025-06-23

**Authors:** Haikun Shang, Yanlei Wei, Shen Zhang

**Affiliations:** Key Laboratory of Modern Power System Simulation and Control & Renewable Energy Technology, Ministry of Education, Northeast Electric Power University, Jilin 132012, China; 2202200205@neepu.edu.cn (Y.W.); 2202300135@neepu.edu.cn (S.Z.)

**Keywords:** transformer, DGA, sample entropy, knowledge distillation, recursive plots, CNNs, CBAM

## Abstract

Dissolved Gas Analysis (DGA) of transformer oil is a critical technique for transformer fault diagnosis that involves analyzing the concentration of gases to detect potential transformer faults in a timely manner. Given the issues of large model parameters and high computational resource demands in transformer DGA diagnostics, this study proposes a lightweight convolutional neural network (CNN) model for improving gas ratio methods, combining Knowledge Distillation (KD) and recursive plots. The approach begins by extracting features from DGA data using the ratio method and Multiscale sample entropy (MSE), then reconstructs the state space of the feature data using recursive plots to generate interpretable two-dimensional image features. A deep feature extraction process is performed using the ResNet50 model, integrated with the Convolutional Block Attention Module (CBAM). Subsequently, the Sparrow Optimization Algorithm (SSA) is applied to optimize the hyperparameters of the ResNet50 model, which is trained on DGA data as the teacher model. Finally, a dual-path distillation mechanism is introduced to transfer the efficient features and knowledge from the teacher model to the student model, MobileNetV3-Large. The experimental results show that the distilled model reduces memory usage by 83.5% and computation time by 73.2%, significantly lowering computational complexity while achieving favorable performance across various evaluation metrics. This provides a novel technical solution for the improvement of gas ratio methods.

## 1. Introduction

Oil-immersed transformers are a critical component of power systems, being responsible for voltage transformation and power transmission. When faults such as overheating or discharge occur, if not promptly identified and handled, they can disrupt the stability of the power system and severely threaten the safety of the power grid [1,2]. Among the transformer fault diagnosis methods, the Dissolved Gas Analysis (DGA) technique has developed rapidly and achieved significant results. DGA assesses the transformer’s operational status and potential fault types by monitoring the composition and concentration changes of dissolved gases in the transformer oil. Typically, the dissolved gas content in transformer oil is stable; however, due to insulation aging, electrical faults, or thermal faults, specific gases such as H_2_, CH_4_, C_2_H_6_, C_2_H_4_, and C_2_H_2_ are produced. The composition and concentration changes of these gases can effectively reflect the type and severity of faults [3,4].

The three-ratio method is widely used in transformer DGA due to its clear principles, well-defined standards, and simplicity of operation [5]. It involves calculating three specific gas ratios which correspond to different types of transformer faults: CH_4_/H_2_, C_2_H_6_/CH_4_, and C_2_H_4_/C_2_H_6_. These ratios are then compared with diagnostic charts or the threshold values specified in the IEC 60599 standard [6] to identify faults such as partial discharges, overheating, arcing, and so on. Through analyzing these gas ratios, the method enables reliable fault classification without requiring complex computations or specialized equipment. It is suitable for preliminary fault screening and routine monitoring in power systems [7]. Reference [8] showed that the three-ratio method can effectively diagnose transformer faults. Reference [9] improved the three-ratio method by combining it with a BP neural network, successfully constructing a transformer fault classification model. Reference [10] extended the application of the three-ratio method using spatial interpolation technology, overcoming its limitations through the application of the Belief Propagation Algorithm (BPA). However, the three-ratio method faces issues such as missing encoding, fuzzy critical values, and gas cross-interference. It is also significantly affected by oil sample aging, environmental interference, and mixed fault gases, which limit its ability to recognize composite faults and early potential defects. Furthermore, the use of a static threshold setting makes it unable to adapt to dynamic operating conditions, leading to poor compatibility with low-concentration gases and new insulating oils, thus limiting its application and reliability in complex scenarios.

To overcome the limitations of the three-ratio method, this study introduces sample entropy (SE) as a feature indicator for transformer fault diagnosis. Multiscale sample entropy (MSE) allows system complexity to be analyzed at different scales in order to provide a comprehensive understanding of signal behavior, thereby improving the sensitivity of fault detection. Higher SE values typically indicate greater system complexity and uncertainty, which may suggest potential faults. Multiscale entropy has been widely applied in various fields, such as biomedical applications [11], mechanical fault detection [12], environmental monitoring [13], financial market analysis [14], speech signal processing [15], image processing [16], and bio-signal analysis [17], and has enhanced early fault prediction capabilities in DGA diagnostics. In recent years, deep learning methods have made significant progress in transformer fault diagnosis due to their powerful feature extraction and nonlinear fitting capabilities [18]. Through constructing deep neural networks, fault features from DGA data can be effectively mined, overcoming the reliance on expert knowledge inherent in traditional methods. Reference [19] proposed an early fault diagnosis method for electro-hydraulic control systems based on residual analysis. The method extracts residual signal features and optimizes the Bayesian network to correct the fault diagnosis results. Reference [20] introduced a concurrent fault diagnosis method for electro-hydraulic control systems based on Bayesian networks and D-S evidence theory. They divided the fault diagnosis approach into multiple sub-models, used OOBNs to establish an initial diagnostic model, and then applied D-S evidence theory for information fusion, improving the reliability of the diagnosis. Reference [21] proposed a ReLU-DBN-based model using unencoded ratios as feature parameters, combined with support vector machines and backpropagation neural networks, thus achieving higher classification performance. Reference [22] proposed a short-term load forecasting method based on LSTM, which improved the transformer load prediction performance by incorporating historical load, holidays, and weather data, providing technical support for solving overload issues.

Despite the significant achievements of deep learning models in transformer DGA diagnostics, their large number of parameters can consume considerable memory and computational resources, affecting their real-time performance. DGA data are provided in one-dimensional form, and converting them into two-dimensional images presents challenges, with traditional conversion methods having limited effectiveness. Recursive plots (RPs) can transform one-dimensional data into two-dimensional images, but conventional convolution operations struggle to extract features effectively. To address this issue, the Convolutional Block Attention Module (CBAM) is introduced, which optimizes feature extraction by incorporating attention mechanisms along both the channel and spatial dimensions, thereby improving fault diagnosis performance and model efficiency.

Knowledge Distillation (KD) is a technique that transfers knowledge from a teacher model to a student model, effectively compressing the model while maintaining high performance [23]. Compared with traditional compression methods, KD offers the advantage of balancing lightweight design with performance, compatibility across architectures, and improved training efficiency; thus, KD provides an optimized path for resource-constrained scenarios such as edge computing and real-time inference. Reference [24] proposes an incremental partial discharge recognition method that combines KD and a graph neural network (GNN). This approach utilizes KD during the incremental training process to prevent model forgetting, while the GNN addresses the issues of small sample sizes and class imbalance, thereby improving the learning performance on new data. Reference [25] introduced a context-aware KD network, which eliminated the gap between the teacher and student networks using an adaptive channel attention mechanism, improving object detection performance and providing an innovative solution for KD applications in object detection. KD not only applies independently for model compression, but also synergizes with deep learning architectures. The Residual Network (ResNet) introduced the residual block structure, successfully addressing the gradient vanishing problem in deep networks and providing a stable foundation for training complex teacher models [26]. Deep learning methods combine feature extraction and fault diagnosis in an end-to-end manner, overcoming the limitations of traditional machine learning methods [27]. Since convolutional layers in deep learning models have weight sharing and local connection properties, they can effectively extract features when processing two-dimensional image data, yielding excellent results. Combining deep learning with KD, using two-dimensional image data for fault diagnosis has become a popular research direction for rapidly identifying potential fault patterns and improving prediction performance.

In summary, the traditional three-ratio method has limitations such as rigid encoding rules, high threshold sensitivity, and insufficient anti-interference ability, which severely restrict its applicability under complex operating conditions. In contrast, although deep learning-based improved methods have significant advantages in terms of feature extraction, they face the dual bottlenecks of high model complexity and limited data representation capability. These limitations highlight the necessity of developing a feature extraction mechanism with high robustness and a lightweight diagnostic architecture.

This study proposes a lightweight model using MSE and KD. First, to address the limitations of traditional three-ratio methods and deep learning approaches in feature extraction and computational efficiency, a feature extraction method based on MSE and RP is introduced, effectively improving the sensitivity and accuracy of transformer fault detection. Secondly, the CBAM is introduced to optimize the feature extraction process of ResNet50, enhancing the model’s ability to focus on key features. Finally, by combining KD technology, a lightweight model is designed that reduces computational resource consumption while maintaining efficient DGA diagnostic performance. The experimental results demonstrate that the proposed model excels in terms of diagnostic accuracy, computational complexity, and storage requirements, providing an efficient and reliable transformer DGA diagnostic solution.

The remainder of this article is structured as follows: Section 2 introduces the principles of the relevant algorithms. Section 3 presents the model based on MSE and KD-CNNs. Section 4 demonstrates the performance of the proposed diagnostic model. Section 5 concludes the study.

## 2. Algorithms and Principles

This chapter introduces the key algorithms and principles underlying the construction of a transformer DGA diagnostic model. Based on transformer DGA data, various algorithms are applied to extract effective features, enabling efficient fault diagnosis. First, the chapter introduces the fault mechanisms and distribution of transformers, followed by an explanation of how MSE improves DGA diagnostic accuracy by analyzing the complexity of data at different time scales. Next, leveraging the features extracted through MSE, the integration of KD and CNN models is discussed. Through KD, the teacher model transfers effective knowledge to the student model, reducing computational load and optimizing model performance. Furthermore, the introduction of RP and CBAM further enhances the feature extraction process. RPs effectively convert one-dimensional data into two-dimensional image features, improving model interpretability, while CBAM uses both spatial and channel attention mechanisms to enable the model to focus on key regions during feature learning.

### 2.1. Transformer Fault Mechanisms and Distribution

Transformer faults result mainly from irreversible damage to insulation materials and conductive components due to thermal stress and electrical discharges. Thermal faults, accounting for 55–60%, are primarily caused by overheating due to issues such as loose connections, core eddy current losses, or winding defects. Electrical faults, making up 35–40%, are triggered by insulation aging, moisture, or mechanical damage.

Faults evolve in stages based on temperature thresholds and gas release characteristics. Thermal faults initially cause local overheating, decomposing oil and cellulose, and thus producing gases such as CH_4_ and C_2_H_6_. As the temperature increases, pyrolysis accelerates, leading to higher concentrations of C_2_H_4_ and H_2_. At critical temperatures, severe overheating generates large amounts of C_2_H_4_ and C_2_H_2_, degrading the insulation. Electrical faults begin with partial discharge, producing H_2_ and CH_4_, and progress to low-energy discharges that release C_2_H_2_ and H_2_. If untreated, high-energy discharges occur, decomposing oil and insulation rapidly, producing gases such as C_2_H_2_, H_2_, CO, and CO_2_, resulting in catastrophic failure.

Unresolved transformer faults pose significant risks. They disrupt operational stability by interfering with voltage regulation, and high-energy discharges can trigger cascading power outages. Faults also pose safety hazards, such as oil leaks, explosions, and fires, which threaten the safety of personnel and infrastructure. Economically, forced shutdowns and replacements may cost millions of dollars, with direct losses exceeding one million dollars for a single large transformer failure. Environmentally, leaked transformer oil can pollute soil and water, and degradation products of insulation materials may violate environmental regulations.

### 2.2. Multiscale Sample Entropy

SE is a widely used tool for measuring the complexity of time series, reflecting the system’s dynamic characteristics by analyzing the self-similarity of the data. However, traditional SE methods typically assess data complexity at a single scale, which may not fully capture the multi-level dependencies present in the data. To address this, this study introduces MSE, aiming to provide a more comprehensive analysis of the time series complexity by examining multiple time scales. By considering the complexity at different scales simultaneously, the MSE method effectively captures both short-term and long-term dependencies, thereby enhancing the overall ability to recognize system behaviors.

#### 2.2.1. Sample Entropy

The core idea behind SE is to measure the complexity of a time series by examining the similarity between adjacent samples. The method involves reconstructing the time series into high-dimensional vectors and evaluating the similarity between these vectors to assess the disorder of the series. SE overcomes its limitations by providing more stable results, particularly for short datasets and noisy signals, making it a robust tool for practical applications.

#### 2.2.2. Algorithm Steps

Given a time series $x=[x_{1},x_{2},\ldots ,x_{N}]$, where *N* is the length of the series, the calculation of SE involves the following steps.

First, the time series *x* is reconstructed into multiple vectors of dimension *m*, where each vector consists of *m* data points. Specifically, each vector is represented as follows:

(1) $$X_{i\;}=[x_{i}\;,x_{i+1}\;,\ldots ,x_{i+m-1}\;],i=1,2,\ldots ,N-m+1$$

where *x*_i_ represents the data points in the time series, *m* is the number of data points in each vector, and *N* represents the total number of data points in the time series.

For any two vectors ***X***_i_ and ***X***_j_, their similarity is measured by the maximum absolute difference between the corresponding elements. If the maximum difference between the two vectors is less than a predefined threshold *r*, the vectors are considered similar. This threshold is usually defined as a multiple of the standard deviation of the time series data.

(2) r=α⋅std(x)

where *r* is the threshold value for similarity, *α* is a constant, typically set to 0.1, and *std*(*x*) is the standard deviation of the time series data *x*.

Next, the number of matches is calculated for each pair of vectors ***X***_i_ and ***X***_j_. A match is defined as a pair of vectors whose maximum absolute difference is less than the threshold *r*.

After obtaining the match counts, the SE is calculated using the following formula:

(3) $$SampEn(m,r,N)=-ln(\frac{A_{m}\;(r)}{B_{m+1}\;(r)\;})$$

where *A*_m_(*r*) is the proportion of vector pairs that are similar when the embedding dimension is *m*, and *B*_m+1_(*r*) is the proportion of vector pairs that are similar when the embedding dimension is *m* + 1.

SE reflects the complexity of the time series by comparing vector similarities at two different dimensions. When the time series exhibits simple structures, the number of similar vector pairs is large, resulting in a small SE value; in contrast, when the time series is more complex, the number of similar vector pairs is small, leading to a higher SE value.

#### 2.2.3. Multiscale Sample Entropy Algorithm Steps

MSE is an extension of the traditional SE that is used to analyze the complexity of time series data across multiple temporal scales. While SE measures the complexity of a time series at a single scale, MSE decomposes the original time series into multiple scales, capturing both short-term and long-term dependencies. This multiscale approach provides a more comprehensive measure of the data’s complexity.

The original time series is first decomposed into multiple time series at different scales by downsampling the data. For each scale τ, downsampling is performed by averaging the data within non-overlapping windows of size τ. The downsampled time series at scale τ is calculated as follows:

(4) $$x_{i}^{\left(\tau \right)}\;=\frac{1}{\tau }\sum_{j=(i-1)\tau +1}^{i\tau }\;x_{j}$$

where τ is the scale factor, and $x_{j}$ represents the data points in the original time series. The new time series $x_{i}^{\left(\tau \right)}$ is the average of the data points within each window of size τ.

Once the time series is decomposed into multiple scales, the traditional SE is calculated for each scale. SE is computed by reconstructing the time series into vectors and measuring the similarity between these vectors. The calculation of SE for each scale follows the same procedure as in the original SE method.

The formula for SE at scale τ is given by

(5) $$SampEn^{\left(\tau \right)}(m,r,N)=-ln\left(\frac{{A_{m}}^{\left(\tau \right)}\;(r)}{{B_{m+1}}^{\left(\tau \right)}\;(r)}\right)$$

where ${A_{m}}^{\left(\tau \right)}\;(r)$ and ${B_{m+1}}^{\left(\tau \right)}\;(r)$ represent the proportions of similar vector pairs at dimensions *m* and *m* + 1, respectively, for the time series at scale τ, with a predefined similarity threshold r.

Finally, the SE values calculated at each scale are averaged to obtain the MSE.

(6) $$MSE(m,r,N)=\frac{1}{S}\;\sum_{\tau =1}^{S}SampEn^{\left(\tau \right)}(m,r,N)$$

where *S* is the number of scales, and $SampEn^{\left(\tau \right)}(m,r,N)$ is the SE calculated at scale *τ*.

### 2.3. Knowledge Distillation

KD is essentially an efficient knowledge transfer method that minimizes the model size by extracting and transferring knowledge from a teacher model to a lightweight student model. Typically, the output of neural networks uses hard labels marked with 0 s and 1 s, which ignore the information from other related classes except the correct one; in contrast, soft labels have values between 0 and 1, which not only indicate the class attribute but also encapsulate implicit information about the relationships between different classes. The teacher model extracts features from labeled training samples and generates high-quality soft labels. These soft labels contain not only the class information of the samples but also reflect the similarity between classes, providing the student model with rich supervisory signals. During the learning process, the student model enhances its prediction ability by mimicking the outputs of the teacher model. This method allows the student model to achieve performance close to or matching that of the teacher model while maintaining a lower complexity.

KD mainly involves the following two key steps.

(1) Soft label generation and distillation temperature. The output of the teacher model is not only the standard hard labels, but can also be smoothed by a temperature coefficient *β* to generate soft labels. The smoothness of the model output is controlled by adjusting *β*. A higher temperature coefficient results in a smoother probability distribution, which provides relational information between classes, allowing the student model to learn more latent feature information, rather than just the classification performance.
(2) Combination of soft labels and hard labels. The training of the student model involves learning not only from the soft labels generated by the teacher model, but also from the real hard labels. To balance the influence of the soft and hard labels during training, a weighting coefficient α is introduced to combine their contributions. The loss function is calculated as follows:
  (7) $$L_{KD}\;=(1-\alpha )L_{hard}\;+\alpha L_{soft}$$
  where *L_KD_* is the total loss function of the student model, *L*_hard_ is the loss associated with the hard labels, *L*_soft_ is the loss associated with the soft labels, and *α* is the coefficient that controls the relative importance of the hard and soft labels. By adjusting the value of *α*, one can control whether the student model focuses more on the hard or soft labels during training.

Since ResNet50 employs a residual block structure, it addresses issues such as vanishing gradients that are common in deep networks. This enables it to effectively extract complex features from the data and generate high-quality soft labels. Therefore, ResNet50 is chosen as the teacher model in this study.

MobileNet, as a network model designed for resource-constrained environments such as mobile devices and embedded systems, utilizes techniques such as depthwise separable convolution and dilated convolution. These techniques improve computational efficiency while reducing the model’s parameter count and computational overhead [28]. Therefore, MobileNet is chosen as the student model in this study.

### 2.4. Convolutional Neural Networks (CNNs)

**Figure 1:**
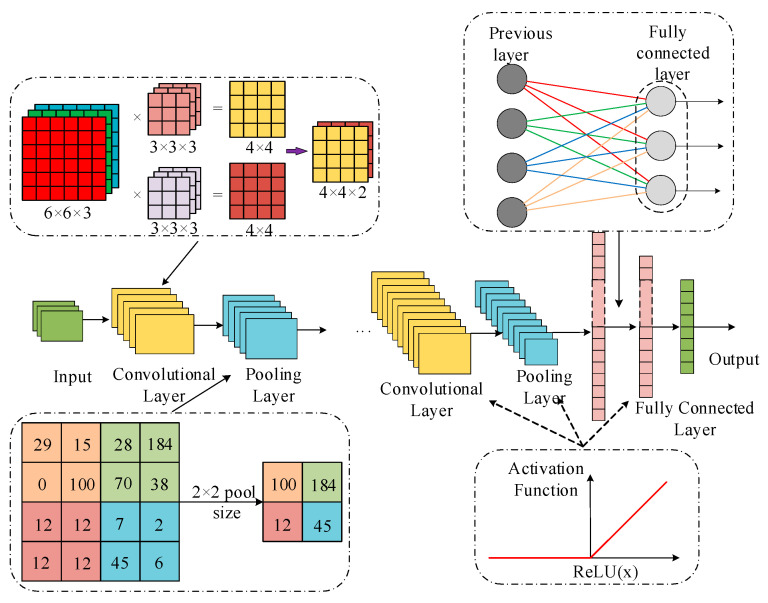
Schematic diagram of the CNN structure.

CNNs are a type of deep learning network that include convolutional operations and have a deep layered structure in a feedforward manner. The structure of a CNN mainly consists of convolutional layers, pooling layers, activation layers, and fully connected layers. The convolutional layer extracts local features from the input data through convolution operations, the pooling layer performs dimensionality reduction on the features, the activation layer introduces nonlinear transformations, and the fully connected layer is used for the final classification or regression task. The specific structure is shown in Figure 1.

**Figure 2:**
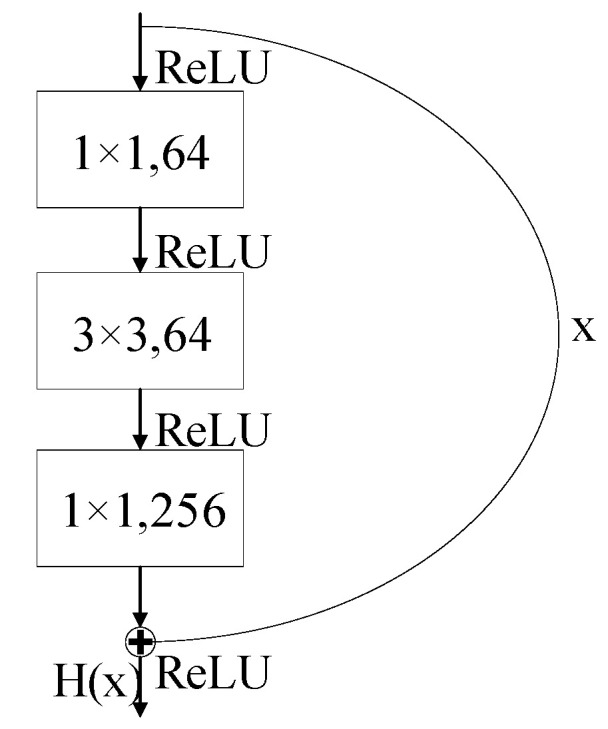
Residual block.

ResNet50 and MobileNet are two popular CNN models. ResNet50 is a deep residual neural network that effectively alleviates the vanishing gradient problem in deep networks through the design of skip connections in its residual blocks. The core residual block in ResNet50 consists of three convolutional layers that form a bottleneck structure, and residual learning is achieved by adding the input features to the output [29]. The network progressively reduces the feature map size through convolutions at different stages, while simultaneously increasing the number of channels, enabling multiscale feature extraction. When the input and output dimensions do not match, shortcut connections adjust the dimensions using 1 × 1 convolutions, ensuring the integrity of the network structure. The structure is shown in Figure 2.

**Figure 3:**
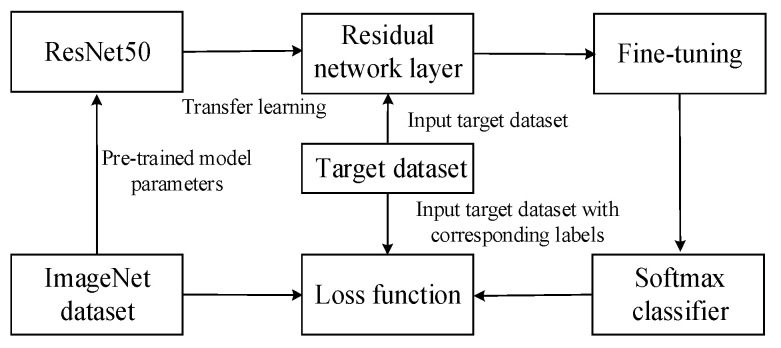
ResNet50 network diagram.

ResNet50, with its powerful feature extraction capabilities, effectively handles high-dimensional and large-scale fault data, identifying complex fault features from them. By introducing residual blocks, gradient propagation through the network is facilitated, thereby accelerating the model’s convergence speed and enhancing the reliability of fault diagnosis. ResNet50 has demonstrated robust performance and strong generalization ability in tasks such as image classification, object detection, and image segmentation, making it a valuable tool in image processing. The specific implementation is shown in Figure 3.

**Figure 4:**
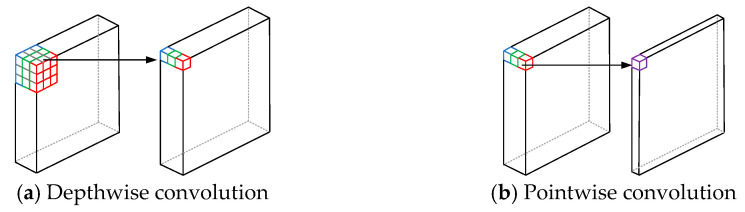
Depthwise separable convolution module.

MobileNet is a lightweight convolutional neural network designed specifically to run on resource-constrained devices. Its core innovation is depthwise separable convolution, which decomposes the traditional convolution operation into two steps: depthwise convolution and pointwise convolution. This greatly reduces computational complexity and the number of parameters. Although MobileNet has a relatively simple structure, it still performs well and is suitable for scenarios with low computational resource requirements. The depthwise separable convolution module is shown in Figure 4.

### 2.5. Recursive Plot

RP is a visualization tool used for analyzing data patterns, designed to visually represent the similarity between data points [30]. In an RP, each data point is mapped to a node in the plot, and the connections between nodes indicate the similarity or dependency between them. The advantage of the RP lies in its ability to capture latent patterns and relationships in the data through the plot’s topological structure without assuming any specific structure for the data. The RP structure is constructed by calculating the similarity between data points, as shown in the process described in Equation (8).

(8) $$R(i,j)=\left\{\begin{array}{l} 1,\;\;if∥x_{i}\;-x_{j}\;∥<\epsilon \\ 0,\;\;if∥x_{i}\;-x_{j}\;∥\geq \epsilon \end{array}\right.$$

In the equation, ***R***(*i*, *j*) represents the similarity between data points *x*_i_ and *x*_j_, and ϵ is the predefined threshold. The Euclidean distance is used to measure the similarity between data points. When the distance between two points is smaller than the threshold, a connection is established between them; otherwise, no connection is formed.

### 2.6. Convolutional Block Attention Module (CBAM)

CBAM includes the channel attention and spatial attention modules [31]. The implementation of channel attention is shown in Equation (9):

(9) $$M_{c}\;=\sigma (FC(ReLU(FC(GlobalPooling(X)))))$$

In the equation, *X* represents the input feature map, *GlobalPooling* denotes the global pooling operation, *FC* is the fully connected layer, and *σ* is the *Sigmoid* function used to generate the weight *M*_c_.

The implementation of spatial attention is shown in Equation (10):

(10) $$M_{s}\;=\sigma (Conv2D(ReLU(Concatenate(MaxPooling(X),AvgPooling(X)))))$$

In the equation, *MaxPooling* and *AvgPooling* represent the max pooling and average pooling operations, respectively. *Conv2D* is the convolution operation, and the spatial attention map is generated through *σ*.

## 3. Transformer DGA Diagnostic Model Based on KD

**Figure 5:**
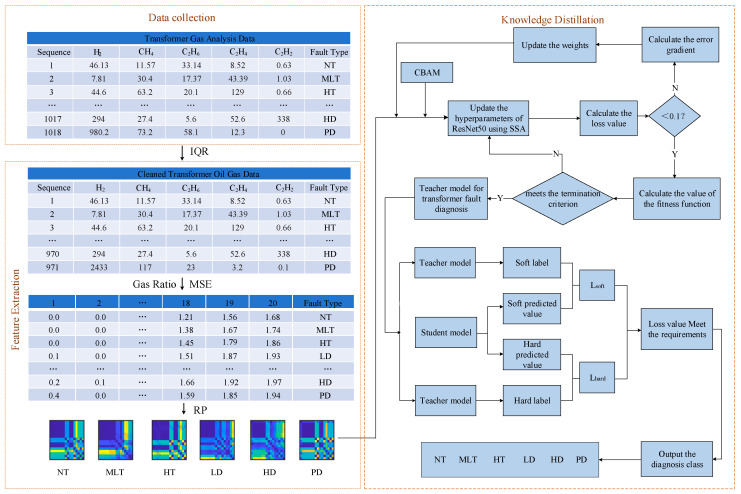
Transformer DGA diagnostic model based on KD.

This study utilizes a model based on KD technology, and the implementation process is as follows:

(1) Data Collection. The dissolved gas data in transformer oil used in this study are sourced from the oil–gas monitoring system. This system collects real-time transformer oil and gas data using multiple sensors.
(2) Data Preprocessing. As the raw data collected on-site may contain outliers that can adversely affect the analysis, this study employs the interquartile range (IQR) method to clean the raw data and remove invalid data points.
(3) Teacher Model Training. The CBAM module is introduced into the convolutional layers of the teacher model, allowing the model to adaptively weight features from different channels and spatial locations. This enables the network to focus on the most representative regions or features in the input image, thereby improving the model’s performance.
(4) Parameter Optimization. The Sparrow Search Algorithm (SSA) [32] is used for hyperparameter optimization of the teacher model, mainly adjusting parameters such as the learning rate and batch size.
(5) Knowledge Distillation. First, the distillation temperature coefficient *β* is set to control the smoothness of knowledge transfer from the teacher model. Then, by adjusting the learning weight *α*, a balance is struck between the soft labels generated by the teacher model and the hard labels. This ensures that the performance of the student model approaches or matches that of the teacher model. The specific implementation block diagram is shown in Figure 5.

As shown in Figure 5, each set of data includes five features, along with the corresponding categorical target that can assume six different values; namely, normal type (NT), medium and low temperature overheating (MLT), high temperature overheating (HT), low-energy discharge (LD), high-energy discharge (HD), and partial discharge (PD).

## 4. Case Study Analysis

To ensure efficient data processing and model training, this study utilized the following computer hardware configurations to meet the computational requirements of the transformer fault diagnosis model experiments. The algorithms used are built on the PyTorch 1.12.0 framework. The CPU manufacturer is AMD, and the GPU manufacturer is NVIDIA. The laptop is a Dell G15 5515, made in China. For detailed information, please refer to Table 1.

### 4.1. Data Collection

Using a chromatograph to detect DGA data has become a key method for effectively diagnosing different types of transformer faults. DGA data are detected using a chromatograph via the following process:

(1) Transformer oil sample collection: Transformer oil samples are randomly extracted from the transformer using specific equipment to ensure the representativeness and accuracy of the sample.
(2) Sample preprocessing: The transformer oil sample undergoes filtration, heating, and other preprocessing operations. These steps remove impurities from the transformer oil and prevent the gas in the oil sample from deteriorating.
(3) Release of dissolved gases: The collected oil sample is placed in a high-temperature environment to release the gases dissolved in the oil. During the heating process, gases in the transformer oil evaporate, forming a gaseous mixture.
(4) Gas separation: The gas sample is separated into different components using the chromatographic column in the chromatograph.
(5) Gas detection and analysis: After separation using the chromatographic column, the gases pass through a detector, where the concentration of each gas is recorded.
(6) Data processing and diagnosis: The detected gas concentration data are input into a computer system for analysis and processing. Finally, by analyzing the changes in the concentration of the gases, the fault type and severity of the transformer are inferred.

The raw data used in this study consist of actual data for gases dissolved in insulating transformer oils from multiple substations, with a total of 1018 samples obtained. Some of the transformer parameters are presented in Table 2.

Each dataset includes five feature variables and their corresponding fault type labels. A sample of the oil chromatographic data is shown in Table 3.

### 4.2. Data Preprocessing

Due to the large volume of transformer oil chromatographic data, the presence of outliers may directly affect subsequent model diagnosis and analysis; therefore, data cleaning, using the IQR method, was first performed. First, the first quartile—the value below which 25% of the data fall—of all numerical columns is calculated. Then, the third quartile—the value below which 75% of the data fall—of all numerical columns is calculated. Finally, the *IQR* is calculated as shown in Equation (11).

(11) $$IQR=Q_{3}-Q_{1}$$

where *IQR* is the interquartile range, which measures the spread between the first and third quartiles; *Q*_1_ is the first quartile; and *Q*_3_ is the third quartile.

To identify potential outliers, the upper and lower boundaries of the dataset are calculated. If a data point is smaller than *Q*_1_ − 1.5 × *IQR*, it is considered a lower outlier; if a data point is larger than *Q*_3_ + 1.5 × *IQR*, it is considered an upper outlier. Following the analysis, 971 valid samples of gas dissolved in transformer oil were obtained. A sample of the oil chromatographic data is shown in Table 4.

Due to the significant differences in gas content values corresponding to different fault types, large differences in feature values during the model training process can affect the experimental results; therefore, this study uses the Min–Max normalization method for normalization. The normalization process is shown in Equation (12).

(12) $$x\prime =\frac{x-\min(x)}{\max(x)-\min(x)}$$

In the equation, *x* represents the sample data, max(*x*) represents the maximum value, and min(*x*) represents the minimum value. The normalized result is represented as *x’*. The data are mapped to the range 0–1 for easier processing. The results are shown in Table 5.

### 4.3. Feature Extraction

As the transformer fault types are closely correlated with the proportions of corresponding gas concentrations, and based on a summary of existing gas ratio methods [33], 17 characteristic gases and their ratios are selected as the analysis targets, as shown in Table 6.

**Table 6:** Seventeen gas ratios selected for analysis.

Sequence	Gas Ratios	Sequence	Gas Ratios	Sequence	Gas Ratios
1	H_2_	7	CH_4_/H_2_	13	CH_4_/TD
2	CH_4_	8	C_2_H_4_/C_2_H_6_	14	(CH_4_ + C_2_H_4_)/TD
3	C_2_H_6_	9	H_2_/ALL	15	(C_2_H_4_ + C_2_H_6_)/TD
4	C_2_H_4_	10	C_2_H_4_/TH	16	(CH_4_ + C_2_H_4_)/TD
5	C_2_H_2_	11	C_2_H_6_/TH	17	(C_2_H_2_ + C_2_H_4_)/H_2_
6	C_2_H_2_/C_2_H_4_	12	C_2_H_2_/TD		

Where TD = CH_4_ + C_2_H_4_ + C_2_H_2_, TH = TD + C_2_H_6_, and ALL = TH + H_2_. Since the fault types are discrete and not directly suitable for input into the model, the classes are converted into numerical values. The corresponding mapping is shown in Table 7.

**Table 7:** Labels corresponding to data types.

Data Type	Label	Data Type	Label
NT	1	LD	4
MLT	2	HD	5
HT	3	PD	6

For the transformer fault classification task, the data labels are encoded and the dataset is divided into training and testing sets for model training and evaluation. The calculated values of some gas ratio data are shown in Table 8.

**Figure 6:**
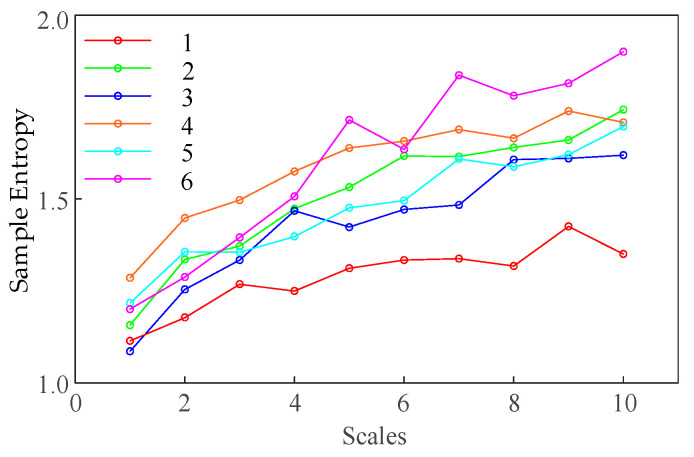
SE values vary with different scales.

To extract more valuable feature information from the DGA data, the MSE method is introduced to extract feature parameters from the raw data. To ensure the stability and effectiveness of the SE calculation, the scale is set to 10, and the calculation is performed on different gas content data. The calculated SE values are shown in Figure 6.

Figure 6 shows that as the scale increases beyond six, the SE values for different transformer faults become relatively uniform, following similar trends. Some fault types exhibit comparable SE values at scale values of four, five, and six; however, at a scale of three, the differences in SE values across various faults become significantly more pronounced. Taking into account that smaller scales may result in the loss of valuable sample information, a scale value of three is selected for this study.

**Figure 7:**
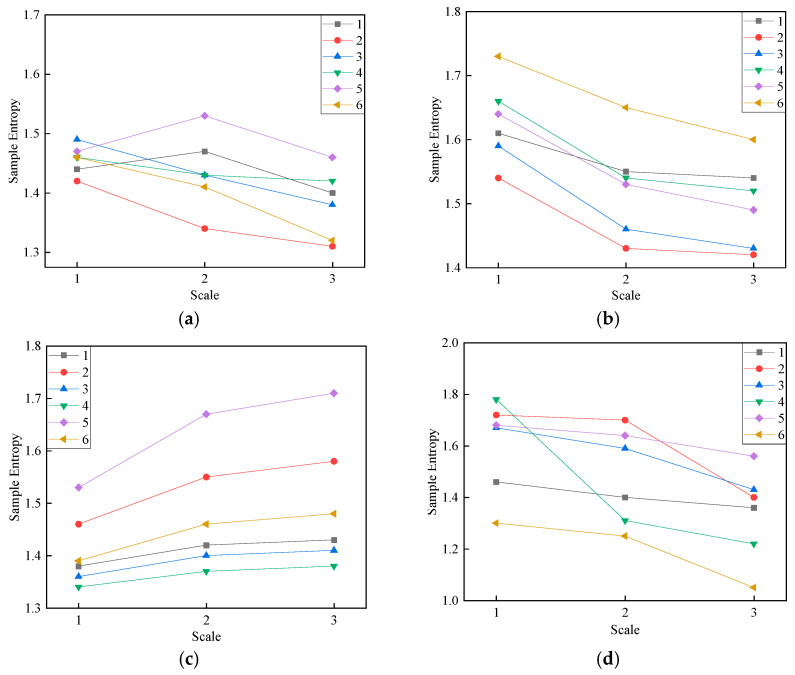
Comparison of different embedding dimensions: (**a**) m = 2; (**b**) m = 3; (**c**) m = 4; (**d**) m = 5.

Taking into account the impact of different embedding dimensions on entropy values, the embedding dimensions range from two to five. Figure 7 shows the comparative results for different fault types under different embedding dimensions.

The impact of the embedding dimension for different fault types is evident from Figure 7. When *m* takes values of two, three, and five, there is a drastic fluctuation in SE values, leading to potential confusion between different fault types. However, when *m* is set to four, the SE values for different fault types exhibit a more gradual change with increasing scales; therefore, *m* is set to four. Partial results are presented in Table 9.

### 4.4. Recursive Plot Generation

**Figure 8:**
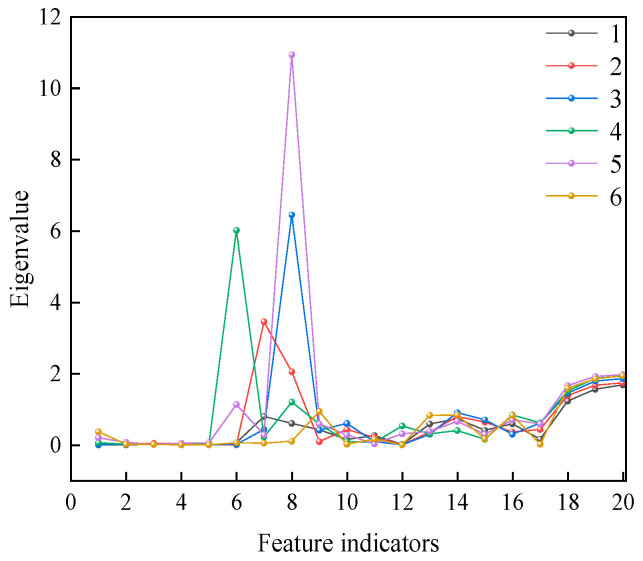
One-dimensional data features.

In deep learning-based DGA research, the effectiveness of feature extraction directly affects model performance. Traditional DGA data face challenges such as high-dimensional coupling, nonlinear dynamics, and weak fault signals. The complex chemical equilibrium relationships between gas concentrations are difficult to represent through conventional features. The 20 data features in Table 6 and Table 7 are plotted as a line graph (Figure 8).

From the one-dimensional data shown in Figure 8, it can be observed that most of the data have a high degree of overlap, making it difficult to extract underlying features. Specifically, the feature values for most data points in different classes are concentrated near the horizontal axis, resulting in low distinguishability between these classes. Therefore, relying solely on these features for classification is unlikely to yield optimal results.

**Figure 9:**
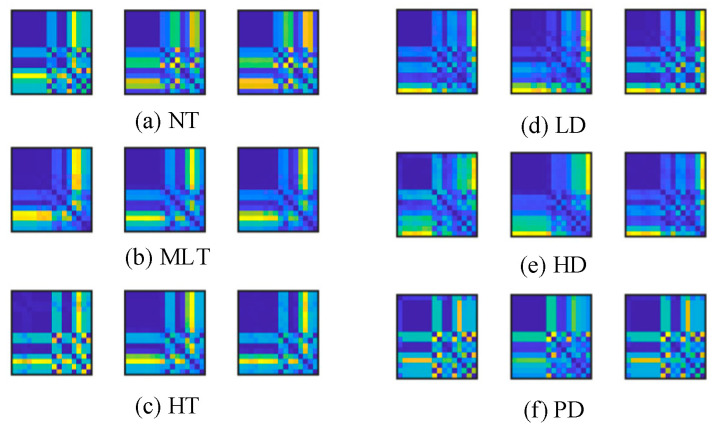
Two-dimensional RPs for analyzing six operating states.

To address the above issue, this study introduces the RP as a feature enhancement tool. The one-dimensional data feature indicators are transformed into two-dimensional recurrence plots, as shown in Figure 9.

The recurrence plots effectively present the multi-dimensional features of the data by mapping multiple feature quantities onto the same plane. They not only clearly identify the underlying relationships within the data but also enhance the visualization, making the analysis process more intuitive and understandable. Figure 9 shows that the distinctions between classes a, b, and c are relatively high, primarily because, during normal transformer operation, the gas concentrations remain stable, with no significant changes. When a thermal fault occurs, the temperature changes gradually; the recurrence plot effectively displays these changes, allowing for differentiation between different fault types. However, as shown in Figure 9, the distinctions between classes d, e, and f are relatively low. This is because the electrical signals cannot be accurately captured by the device during electrical faults, leading to less pronounced differentiation in the recurrence plots, which in turn affects fault type classification.

The recursive matrix texture pattern can explicitly express the chaotic characteristics and critical points of mutation in gas concentration sequences, enhancing the discriminative ability between different classes and providing auxiliary feature representations with physical interpretability for deep neural networks.

### 4.5. SSA-Optimized Hyperparameters for ResNet50

#### 4.5.1. Hyperparameter Selection for ResNet50

The selection of hyperparameters is crucial for improving network performance during the model training process. The hyperparameters for the ResNet50 model, including the learning rate, decay factor, step size, and batch size, are initially set based on empirical judgment. The initial values of these hyperparameters are shown in Table 10.

**Figure 10:**
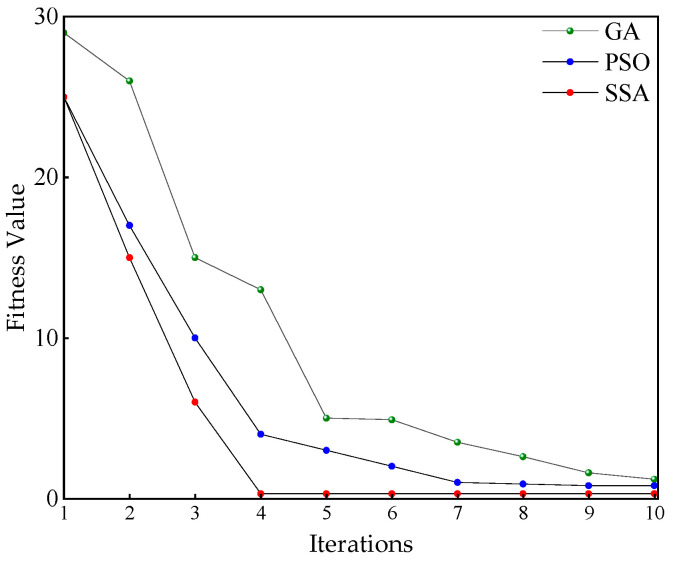
Fitness comparisons.

Inappropriate hyperparameter configurations may result in a learning rate that is either too small or too large, leading to overfitting or underfitting, which negatively impacts the model’s generalization ability and results in unsatisfactory training performance. Additionally, an unreasonable batch size can cause slow training speed or waste of computational resources; in particular, issues such as memory overflow may occur with excessively large batch sizes. Furthermore, an improper decay factor and step size settings may lead to instability in the optimization process, manifested as gradient oscillation or stagnation in convergence, thereby affecting the effective training of the model. To address this, this study employs the SSA for hyperparameter optimization. This algorithm simulates the foraging behavior of sparrow populations and possesses strong global search capabilities. A comparison is also made with the Genetic Algorithm (GA) and Particle Swarm Optimization (PSO). The maximum training iterations are set to 10, with 20% of the population representing the discoverers. The response value changes for GA, PSO, and SSA over 10 iterations are shown in Figure 10.

As shown in Figure 10, in the early stages of iteration, the SSA quickly escapes local optima through extensive search and random exploration, gradually converging towards the global optimum. As the iterations progress, the search range of SSA narrows, avoiding the dramatic fluctuations observed in the GA and PSO searches and ensuring that the fitness value steadily and rapidly approaches the optimal solution. The optimized hyperparameters for ResNet50 are finally obtained, as shown in Table 11.

#### 4.5.2. Model Architecture and Attention Mechanism

**Figure 11:**
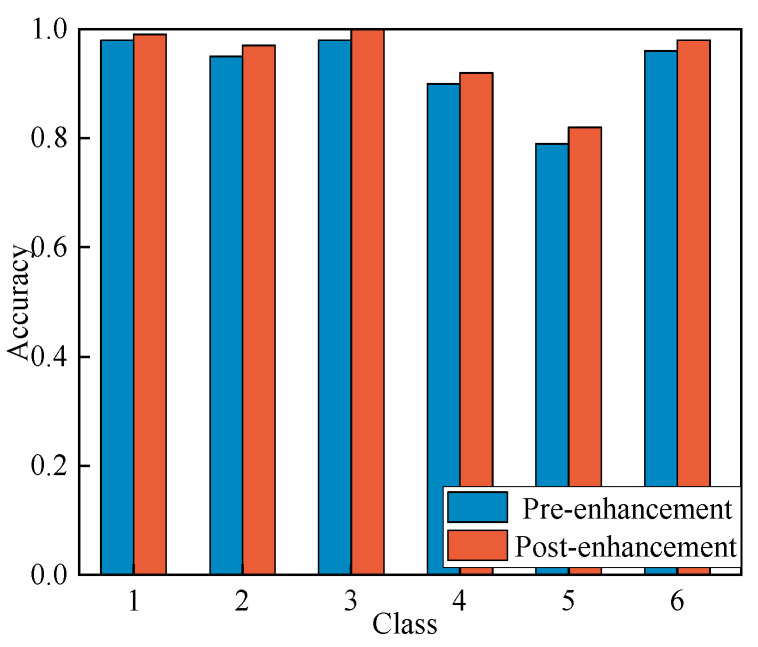
Comparison of accuracy before and after model enhancement.

In this study, the CBAM attention module is introduced to enhance the model’s performance. The comparison results are shown in Figure 11.

As shown in Figure 11, the model with the CBAM attention enhancement demonstrates performance improvements across all classes. The enhanced model exhibits significant performance gains in each class, particularly in class 5, where a noticeable performance boost is observed. The data indicate that the incorporation of the CBAM attention mechanism results in a 2.05% overall performance improvement on the test set, validating the effectiveness of this approach.

### 4.6. Analysis of Teacher Model Results

**Figure 12:**
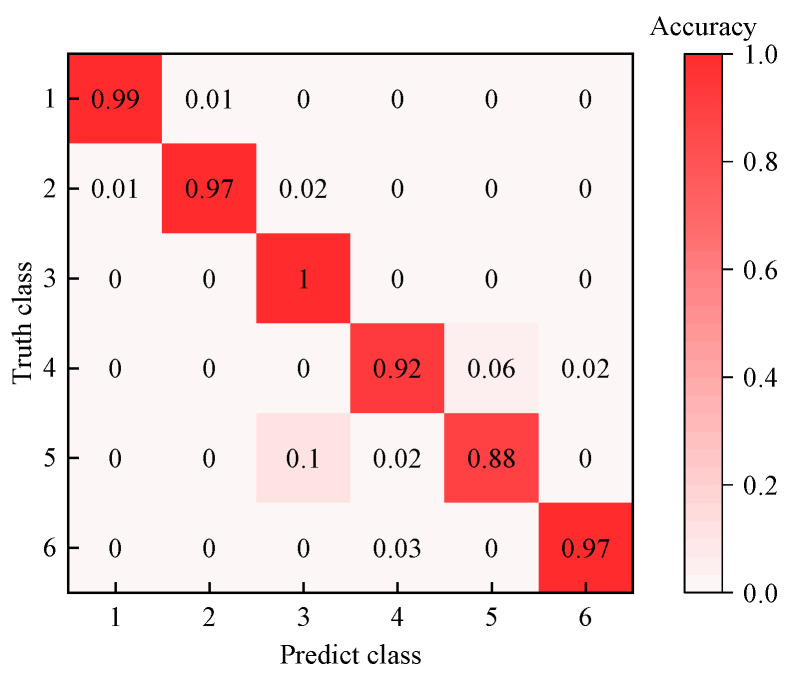
Confusion matrix for the ResNet50 model.

The classification performance of the ResNet50 model on the test set is obtained by simulating the feature images of transformer oil–gas data after data cleaning, as shown in Figure 12.

As shown in Figure 12, the model exhibits significant differences in recognition performance across various categories. The recognition rate reaches 99% under normal operating conditions, demonstrating excellent diagnostic capability. The recognition performance for overheating categories exceeds 97%, indicating that the model effectively captures temperature variation features and can promptly detect potential anomalies with high precision. For discharge classes, the recognition rates of low-energy discharge (92%) and partial discharge (97%) are relatively high, suggesting that the model efficiently captures their characteristics for precise identification. However, the recognition performance for high-energy discharge is relatively lower at 88%, primarily due to its strong thermal effects, causing feature overlap with the high-temperature overheating category. Despite this recognition bias, high-energy discharge still achieves a 100% detection rate as a severe condition.

### 4.7. Distillation Hyperparameter Selection

In KD, the temperature coefficient *β* and learning weight *α* play crucial roles in the distillation process. Both parameters need to be adjusted during the training process to determine the optimal combination. In this study, MobileNetV3-Large is selected as the student model for the distillation operation.

**Figure 13:**
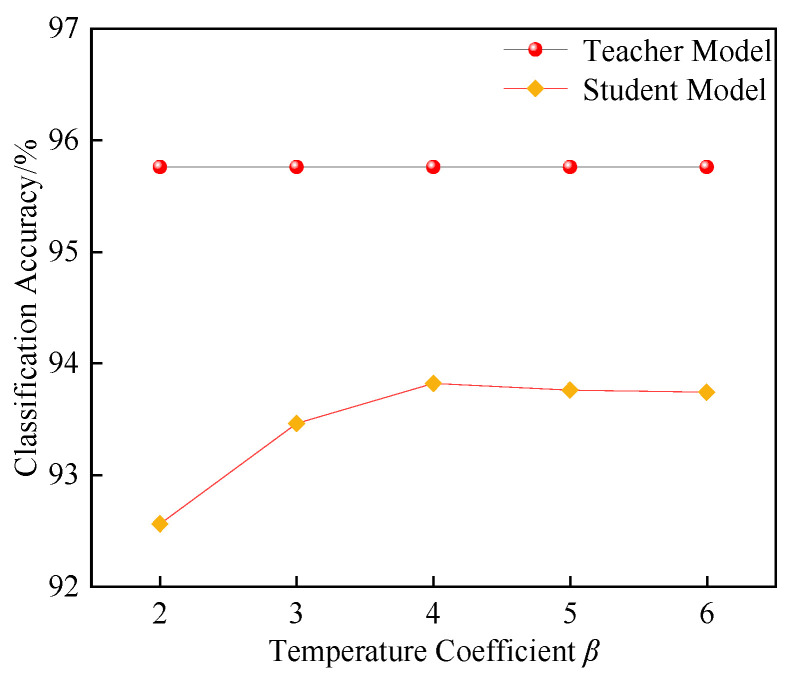
Comparison under different *β* values.

The setting of the temperature coefficient *β* affects the smoothness of the class output probability distribution of the teacher model, making the distribution either flatter or sharper. In this study, with *α* = 0.5, the change in recognition performance at different values of *β* is analyzed, as shown in Figure 13.

As shown in Figure 13, the prediction performance of the student model improves as *β* is increased from two to four. This indicates that as *β* increases, the output probability distribution of the teacher model becomes smoother, reducing the differences between classes, which allows the student model to learn more latent information from the teacher model. However, as *β* increases from four to six, the prediction performance decreases, indicating that an excessively high *β* value causes the teacher model’s output to become too smooth. This leads the student model to overly rely on the teacher model’s soft labels, suppressing the learning of the true labels and, consequently, affecting classification performance. Therefore, this study selects *β* = 4, with the classification shown in Figure 13. The prediction performance of the student model improves as *β* increases from two to four, reaching 93.82%, at which the student model achieves the optimal classification performance. This setting strikes the best balance between the information transfer of the teacher model’s soft labels and the true labels, leading to the best classification results.

**Figure 14:**
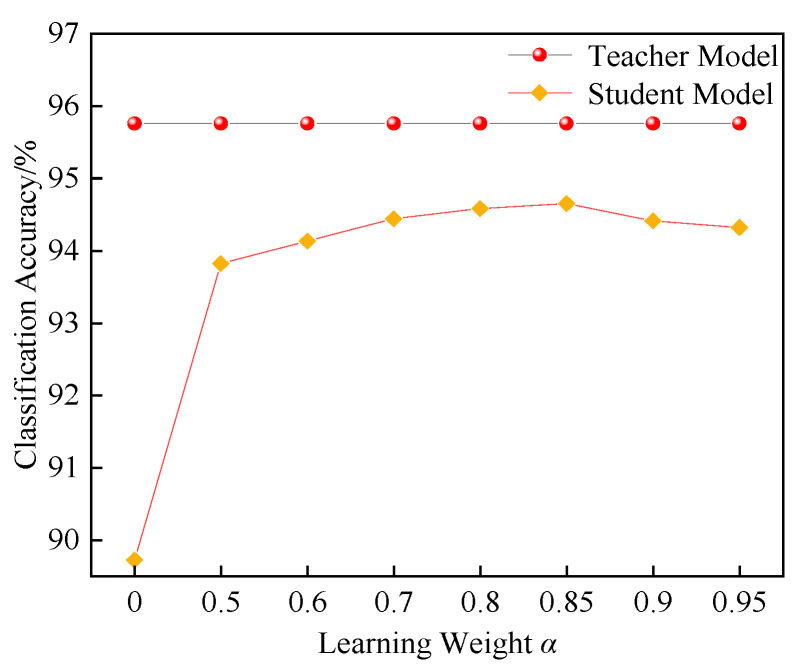
Comparison under different *α* values.

The network learning weight *α* determines the degree to which the student model relies on the labels. When *α* is large, the student model relies more on the soft labels output by the teacher model; in contrast, when *α* is small, the student model depends more on the true labels. Therefore, the choice of *α* directly affects the learning weight between the soft labels and the true labels for the student model. To find an appropriate *α* value, this study conducts KD simulation experiments with MobileNetV3-Large as the student model and determines the network training performance for different *α* values, as shown in Figure 14.

As shown in Figure 14, when the *α* parameter is set to 0.85, the classification performance of the student model reaches 94.65%, demonstrating good classification performance. This indicates that, with *α* = 0.85, the student model can effectively balance the influence of the true labels and the teacher model’s soft labels during the learning process, thereby improving classification performance. However, as the learning weight *α* continues to increase, the classification performance does not improve further and instead stabilizes, suggesting that increasing *α* does not significantly enhance the learning performance of the student model. This may lead to the model becoming overly dependent on the teacher model’s soft labels, while neglecting the importance of the true labels; therefore, this study selects *α* = 0.85.

### 4.8. Analysis of Student Model Comparison Results

**Figure 15:**
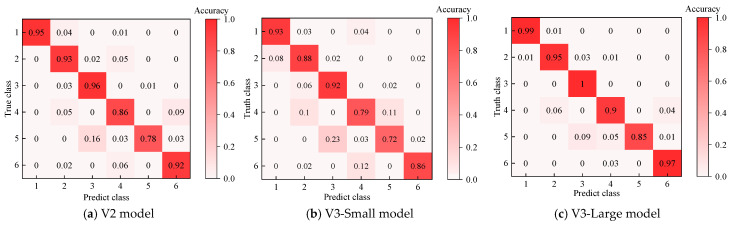
Confusion matrices for recognition results.

MobileNetV2 (abbreviated as V2), MobileNetV3-Small (abbreviated as V3-Small), and MobileNetV3-Large (abbreviated as V3-Large) are selected as student models for comparative analysis. Since these three student models have similar structures, the hyperparameters are set uniformly as *α* = 0.85 and *β* = 4, and the data are divided into a training set and a test set in an 8:2 ratio. KD is performed using the training set, with the teacher model’s output guiding the training of the student model. The performances of the student models are then evaluated using the test set, and confusion matrices are generated. The specific results are shown in Figure 15, where 1–6 represent different transformer fault types.

As shown in Figure 15, the V3-Large model consistently outperforms the other two models in classification performance across all six classes; specifically, the classification performance of the V2 model falls between those of the V3-Large and V3-Small models, with the V3-Small model exhibiting the lowest performance. In the case of MLT faults, the V3-Small model misclassified the fault as a normal state with a probability of 8%, which poses a significant risk to the stable operation of the transformer. The V3-Large model demonstrates a clear advantage in all test classes, with stable classification performance. This indicates that the V3-Large model has stronger learning and recognition capabilities in classification tasks, effectively improving the model’s reliability.

### 4.9. Comparative Analysis

To validate the effectiveness of the proposed method, ResNet50 was selected as the teacher model, while V2, V3-Small, and V3-Large were used as student models for analysis. To evaluate the overall performance of the diagnostic model, the accuracy, recall, precision, and F1 score were chosen as the evaluation metrics to assess the model’s diagnostic effectiveness.

**Figure 16:**
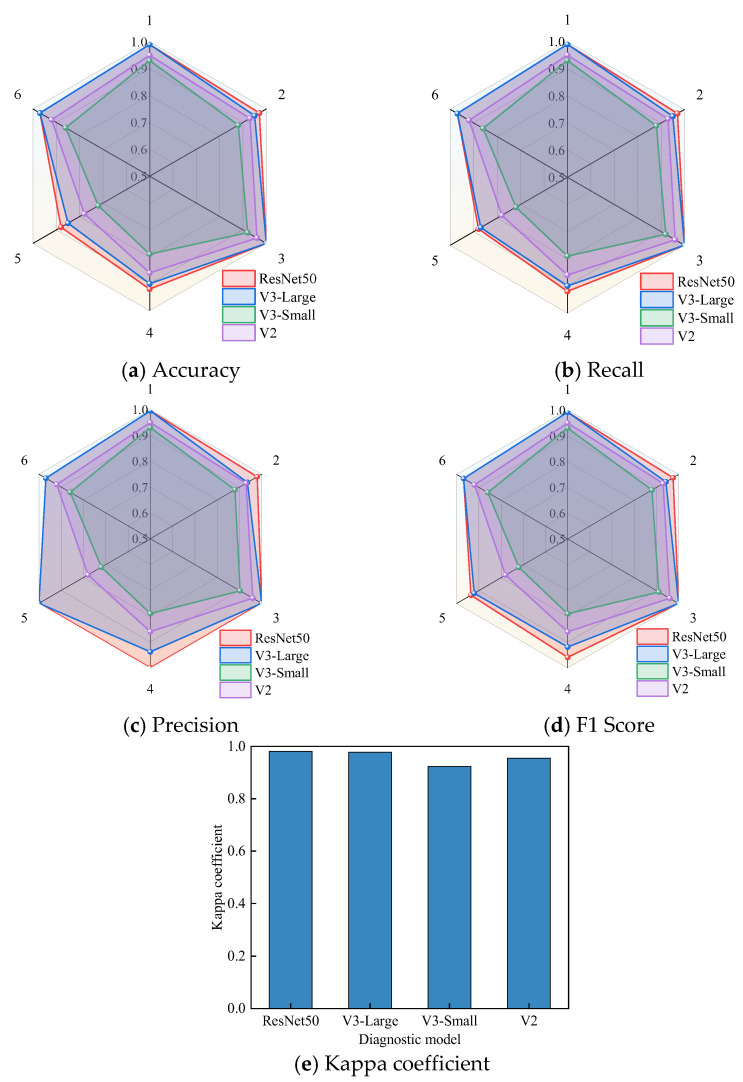
Diagnosis results.

The results of the different metrics are shown in Figure 16.

Figure 16a demonstrates that V3-Large exhibits markedly superior predictive performance in class 1 and class 3 relative to V3-Small and V2, with its discriminative capability approaching the optimal range for these readily distinguishable fault types. In classes 4 and 5, despite the elevated diagnostic complexity, V3-Large maintains a clear performance advantage over other student models and shows close correspondence to the teacher model’s performance benchmarks. Figure 16b shows that V3-Large has a recall rate of 0.94 in class 4, which is significantly better than the other models. This indicates that it can effectively capture more true positives, reduce false negatives, and further improve sensitivity. The recall rate of V3-Large is also better for other classes, further confirming its strong capability for different classes. Figure 16c shows that V3-Large achieves precisions of 0.99 and 1.00 in class 1 and class 3, respectively, demonstrating its exceptionally high precision and low false positive rate in diagnosing these two fault types. Figure 16d shows that the F1 score of V3-Large is the highest among all fault types, with an especially impressive F1 score of 0.94 in classes 4 and 5. The F1 score remains stable, consistently above 85%, indicating that V3-Large can effectively balance precision and recall, exhibiting strong generalization ability. Finally, the results in Figure 16e show that the Kappa coefficient of V3-Large is 97.8%, which is close to the Kappa coefficient of the ResNet50 teacher model (98.1%), indicating that V3-Large’s performance in transformer DGA diagnostics is highly consistent with the teacher model, demonstrating strong agreement.

After KD, the memory usage and diagnostic time of each model are shown in Table 12.

As shown in Table 12, the memory usage of ResNet50 is 98 MB, while the memory usage of MobileNetV3-Large is only 16.2 MB, representing an 83.5% reduction in memory usage and a 73.2% decrease in computation time. This demonstrates that KD effectively reduces the memory usage of the student model.

## 5. Conclusions

This study proposed a lightweight convolutional neural network model for transformer DGA diagnostics that combines MSE for feature extraction, KD, and enhanced feature RPs. The conclusions are as follows:

1. Feature Extraction: First, the ratio method was used to process five types of characteristic gases, resulting in 17 features. Simultaneously, MSE was applied to analyze the data, with an embedding dimension of four and a scale of three, yielding three entropy values. These entropy values were then converted into RPs. After integration and processing, 20 features were obtained, effectively increasing the number of features and providing richer and more effective feature information for subsequent fault diagnosis.
2. Hyperparameter Optimization and Attention Mechanism: In this study, SSA was used to optimize the hyperparameters of the ResNet50 model, and a comparative analysis with PSO and GA was conducted, confirming the superiority of the SSA optimization method in parameter adjustment. Additionally, the CBAM attention enhancement module was introduced, enabling the model to more effectively extract key features from the transformer oil and gas data.
3. Knowledge Distillation Technique: In the framework of the teacher model ResNet50, and the student model MobileNetV3-Large, this study introduced a dual-route KD mechanism for transferring the efficient features and knowledge from the teacher model to the student model. The simulation results demonstrated that the knowledge-distilled V3-Large model has only 16.5% of the parameters of ResNet50, reduces the computation time by 73.2%, and outperforms other methods in multiple performance metrics, including the accuracy, recall, precision, F1 score, and Kappa coefficient. These results validate the effectiveness and superiority of the proposed model.

In the future, the authors will attempt to collect more on-site transformer fault data to further validate the effectiveness and practicality of the proposed model.

## Figures and Tables

**Table 1:** Computer configuration information.

Environment Configuration	Configuration Value
CPU	AMD Ryzen 7 5800H
GPU	NVIDIA GeForce RTX 3060
Operating System	Windows 10
Python	3.10
CUDA	11.6
Pytorch	1.12.0 + cu116

**Table 2:** Transformer parameters.

Type	Parameters
Substation Voltage Level	750 kV
Transformer Model	ODFPS-500000/750
Production Time	June 2018
Commissioning Date	14 May 2019
Main Transformer Cooler Model	YF-400

**Table 3:** Transformer oil chromatographic data.

Sequence	H_2_	CH_4_	C_2_H_6_	C_2_H_4_	C_2_H_2_	Fault Type
1	46.13	11.57	33.14	8.52	0.63	NT
2	7.81	30.4	17.37	43.39	1.03	MLT
3	44.6	63.2	20.1	129	0.66	HT
4	30	62	60	460	3.4	LD
…	…	…	…	…	…	…
1017	294	27.4	5.6	52.6	338	HD
1018	980.2	73.2	58.1	12.3	0	PD

**Table 4:** Transformer oil chromatographic data after data cleaning.

Sequence	H_2_	CH_4_	C_2_H_6_	C_2_H_4_	C_2_H_2_	Fault Type
1	46.13	11.57	33.14	8.52	0.63	NT
2	7.81	30.4	17.37	43.39	1.03	MLT
3	44.6	63.2	20.1	129	0.66	HT
4	30	62	60	460	3.4	LD
…	…	…	…	…	…	…
970	294	27.4	5.6	52.6	338	HD
971	2433	117	23	3.2	0.1	PD

**Table 5:** Normalized results.

Sequence	H_2_	CH_4_	C_2_H_4_	C_2_H_6_	C_2_H_2_	Fault Type
1	0.017	0.007	0.014	0.003	0	NT
2	0.003	0.018	0.007	0.014	0.001	MLT
3	0.016	0.038	0.008	0.043	0	HT
4	0.011	0.037	0.025	0.153	0.002	LD
…	…	…	…	…	…	…
970	0.106	0.106	0.002	0.018	0.180	HD
971	0.878	0.07	0.009	0.001	0	PD

**Table 8:** Partial gas ratios.

1	2	3	4	5	6	7	8	9	10	11	12	13	14	15	16	17	Type
0.4	0.0	0.0	0.0	0.0	0.1	0.1	0.1	0.9	0.0	0.2	0.0	0.8	0.8	0.2	0.8	0.0	6
0.0	0.0	0.0	0.0	0.0	0.0	1.4	6.4	0.2	0.6	0.1	0.0	0.3	0.9	0.7	0.3	0.6	3
0.2	0.1	0.0	0.1	0.1	1.1	0.3	10.9	0.6	0.3	0.0	0.3	0.4	0.7	0.3	0.7	0.6	5
0.1	0.0	0.0	0.0	0.0	6.0	0.2	1.2	0.6	0.1	0.1	0.5	0.3	0.4	0.2	0.8	0.6	4
0.0	0.0	0.0	0.0	0.0	0.1	0.8	0.6	0.4	0.2	0.3	0.0	0.6	0.7	0.4	0.6	0.2	1
…	…	…	…	…	…	…	…	…	…	…	…	…	…	…	…	…	…
0.0	0.0	0.0	0.0	0.0	0.0	3.5	2.1	0.1	0.4	0.2	0.0	0.4	0.8	0.6	0.4	0.4	2

**Table 9:** Results of partial multiscale entropy values.

Scale 1	Scale 2	Scale 3	Data Type
1.21	1.56	1.68	NT
1.38	1.67	1.74	MLT
1.45	1.79	1.86	HT
1.51	1.87	1.93	LD
…	…	…	…
1.66	1.92	1.97	HD
1.59	1.85	1.94	PD

**Table 10:** ResNet50 hyperparameters.

Hyperparameters	Value	Range
learning rate	0.0001	0.00001~0.001
decay factor	0.1	0.1~0.9
step size	4	1~10
batch size	64	4~256

**Table 11:** Corresponding SSA–ResNet50 hyperparameters.

Hyperparameter	Value
learning rate	0.00008
decay factor	0.86
step size	2
batch size	32

**Table 12:** Memory usage and diagnosis times of different models.

Model	Memory Usage (MB)	Diagnosis Time (s)
ResNet50	98.0	15.3
MobileNetV3-Large	16.2	4.1
MobileNetV3-Small	7.9	2.3
MobileNetV2	14.0	3.9

## Data Availability

The data that support the findings of this study are not publicly available due to the confidentiality requirements of one ongoing project.

## Abbreviations

$x_{j}$	the data points in the original time series
${x_{i}}^{\left(\tau \right)}$	the average of the data points within each window of size
*L*_hard_	the loss associated with the hard labels
*L*_soft_	the loss associated with the soft labels
*R*(*i, j*)	the similarity between data points *x*_i_ and *x*_j_
*X*	the input feature map
*Q*_1_	the first quartile
*Q*_3_	the third quartile
*x*	the sample data
max(*x*)	the maximum value
min(*x*)	the minimum value
α	learning weights
β	temperature coefficient
τ	scale factor
ϵ	the predefined threshold
σ	the *Sigmoid* function used to generate the weight *M*_c_
DGA	Dissolved Gas Analysis
KD	Knowledge Distillation
SE	sample entropy
MSE	Multiscale sample entropy
CBAM	Convolutional Block Attention Module
ResNet	Residual Network
MobileNet	Mobile Neural Network
BPA	Belief Propagation Algorithm
IGWO	Improved Grey Wolf Optimization
WELM	Weighted Extreme Learning Machine
RP	recursive plots
CNN	convolutional neural network
GNN	graph neural network
SSA	Sparrow Optimization Algorithm
IQR	interquartile range
GA	Genetic Algorithm
PSO	Particle Swarm Optimization
NT	normal type
MLT	medium and low temperature overheating
HT	overheating
LD	low-energy discharge
HD	high-energy discharge
PD	partial discharge
